# The *Bacillus cereus* Strain EC9 Primes the Plant Immune System for Superior Biocontrol of *Fusarium oxysporum*

**DOI:** 10.3390/plants11050687

**Published:** 2022-03-02

**Authors:** Kenneth Madriz-Ordeñana, Sercan Pazarlar, Hans Jørgen Lyngs Jørgensen, Tue Kjærgaard Nielsen, Yingqi Zhang, Kai Lønne Nielsen, Lars Hestbjerg Hansen, Hans Thordal-Christensen

**Affiliations:** 1 Department of Plant and Environmental Sciences and Copenhagen Plant Science Centre, Section for Plant and Soil Science, University of Copenhagen, 1871 Frederiksberg, Denmark; sercan.pazarlar@ege.edu.tr (S.P.); hjo@plen.ku.dk (H.J.L.J.); zhangyingqi@nwafu.edu.cn (Y.Z.); htc@plen.ku.dk (H.T.-C.); 2 Department of Plant and Environmental Sciences and Copenhagen Plant Science Centre, Section for Microbial Ecology and Biotechnology, University of Copenhagen, 1871 Frederiksberg, Denmark; tkn@plen.ku.dk (T.K.N.); lhha@plen.ku.dk (L.H.H.); 3 Knud Jepsen a/s, 8382 Hinnerup, Denmark; kai@queen.dk

**Keywords:** biological control agents, induced resistance, defence priming, antimicrobial secondary metabolites, *Kalanchoe blossfeldiana*

## Abstract

Antibiosis is a key feature widely exploited to develop biofungicides based on the ability of biological control agents (BCAs) to produce fungitoxic compounds. A less recognised attribute of plant-associated beneficial microorganisms is their ability to stimulate the plant immune system, which may provide long-term, systemic self-protection against different types of pathogens. By using conventional antifungal in vitro screening coupled with *in planta* assays, we found antifungal and non-antifungal *Bacillus* strains that protected the ornamental plant Kalanchoe against the soil-borne pathogen *Fusarium oxysporum* in experimental and commercial production settings. Further examination of one antifungal and one non-antifungal strain indicated that high protection efficacy *in planta* did not correlate with antifungal activity *in vitro*. Whole-genome sequencing showed that the non-antifungal strain EC9 lacked the biosynthetic gene clusters associated with typical antimicrobial compounds. Instead, this bacterium triggers the expression of marker genes for the jasmonic and salicylic acid defence pathways, but only after pathogen challenge, indicating that this strain may protect Kalanchoe plants by priming immunity. We suggest that the stimulation of the plant immune system is a promising mode of action of BCAs for the development of novel biological crop protection products.

## 1. Introduction

The biological control of soil-borne plant diseases involving beneficial microorganisms has gained considerable attention as an alternative to synthetic fungicides that are environmentally problematic and difficult to use against these pathogens [1,2,3]. These BCAs can positively influence fitness [4,5,6,7,8] and protect plants from pathogen infection by competition for space and nutrients, by parasitism and by antibiosis [9,10]. The latter mechanism has been widely considered in the development of biofungicides, taking advantage of the ability of the BCAs to produce toxic compounds directly targeting fungal pathogens [11,12,13]. Endospore-forming bacteria include interesting beneficial microorganisms since they can survive as dormant spores, which is an advantage for the formulation of biological control products [14]. Typically, *Bacillus* sp. have been screened for their ability to produce an array of fungi-toxic compounds [13,15,16,17]. Although less exploited as a mechanism for disease control, plant-associated microorganisms can also benefit plants by boosting their plant immune system through a phenomenon called induced resistance (IR), which may provide long-term, systemic protection against different types of pathogens [18,19,20]. Two main and interrelated mechanisms are generally recognised as part of IR: (1) systemic acquired resistance (SAR), which is associated with salicylic acid (SA) signalling and accompanied by upregulation of genes encoding certain pathogenesis-related (PR) proteins; and (2) induced systemic resistance (ISR), which is associated with jasmonic acid (JA) signalling and upregulation of genes encoding, e.g., plant defensins and lipoxygenases (*LOX*) [21,22]. A specific type of IR is known as defence priming. Here, the primed plant shows no apparent defence activity in the absence of infection, but it is predisposed to respond faster and stronger upon pathogen attack, even weeks after defence priming was triggered [19,23,24]. The mechanisms underlying defence priming are believed to include epigenetic chromatin modification, such as DNA hypomethylation, increased histone 3 activation marks and reduced histone 3 silencing marks [25,26]. Notably, plant-associated bacteria may activate defence priming [27,28,29,30], with no or low energy expenses in the absence of pathogen attack [31,32]. This makes these microorganisms attractive BCAs that promote plant self-defence with low energy cost.

In this study, we searched for endospore-forming bacteria with potential as BCAs to control the soil-borne fungal pathogen *Fusarium oxysporum* [33] affecting the greenhouse production of Kalanchoe (*Kalanchoe blossfeldiana* Poelln.) and many other crops. The fungus penetrates through roots to invade and obstruct the vascular tissue, causing necrosis and wilting. We screened for disease-protecting bacterial strains using conventional antifungal in vitro screening coupled with *in planta* assays. We first identified antifungal (AF) and non-antifungal (NAF) bacterial strains as determined by in vitro confrontation tests against *F. oxysporum*. Assays *in planta* allowed us to select both AF and NAF strains that conferred high levels of protection against this pathogen under greenhouse experimental settings as well as in pilot trials under commercial production conditions. The results indicated that the ability of selected bacterial strains to protect against *F. oxysporum* infection *in planta* did not correlate with strong antifungal activity *in vitro*. We provide whole-genome sequence data indicating that a disease-protecting NAF strain EC9 lacks biosynthetic gene clusters (BGCs) for known antimicrobial compounds. We present evidence indicating that the bacterial-mediated activation of plant defence provided strong protection against *F. oxysporum*. Since the application of the strain alone did not cause the activation of defence-related genes, protection is suggested to involve priming, which has great potential in the development of novel biological crop protection products.

## 2. Results

### 2.1. Initial Selection of Strains

A total of 390 endospore-forming bacterial strains were isolated from Kalanchoe material. Preliminary screening of these strains for fungal growth-inhibiting activity *in vitro*, here defined as antifungal (AF) activity against *F. oxysporum* isolate CP2321, was performed by the visual observation of fungal growth inhibition zones between the fungal and bacterial colonies on PDA plates. Strains that showed weak or no evident AF activity were defined as non-antifungal (NAF). Some of these NAF strains conferred protection in preliminary *in planta* assays (data not shown). We selected seven putative *in planta*-protecting strains, including three AF and four NAF strains, for further testing. For clarity, AF strains are depicted with orange colour in the graphs, whereas NAF strains are represented in blue colour.

### 2.2. Treatment of Kalanchoe Plants with Selected Strains Confer Protection against F. oxysporum

**Figure 1:**
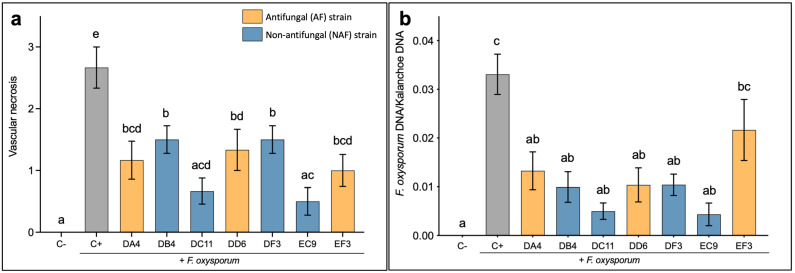
*In planta* evaluation of seven selected bacterial strains in Kalanchoe. (**a**) Scoring of the extent of vascular necrosis in Kalanchoe stems. The scores relate to the percentage of necrotic vascular tissue as described in Materials and Methods. (**b**) Quantification of the relative amount of *Fusarium oxysporum* DNA in Kalanchoe stems. C− is mock treatment (water) and C+ is untreated inoculated control plants. Error bars represent the standard error of the mean. *n* = 6. Treatment means marked with different letters are significantly different at *p* ≤ 0.05.

The seven selected strains were further evaluated *in planta* for their ability to confer protection against *F. oxysporum* infection. A general reduction in the extent of vascular necrosis in Kalanchoe stems was observed at the crown level following treatment with all the tested strains in comparison to the untreated inoculated (C+) control plants (Figure 1a). The NAF strains DC11 and EC9 showed the lowest vascular necrosis score. Notably, for these two strains, the VN score was not significantly different from the uninoculated control (C−) plants. The quantification of the relative amount of fungal DNA in the vascular tissue (Figure 1b) correlated to some extent with the VN score. Thus, treatment with the strains, except for strain EF3, resulted in a significant reduction in *F. oxysporum* DNA in the stems compared to the untreated, inoculated control. Additionally, there was no significant difference compared to the uninoculated control plants. Interestingly, the best protection was seen for NAF strains DC11 and EC9, both for vascular necrosis and for the quantification of fungal DNA (Figure 1a,b).

### 2.3. Protection In Planta against F. oxysporum Does Not Correlate with High Antifungal Activity In Vitro

**Figure 2:**
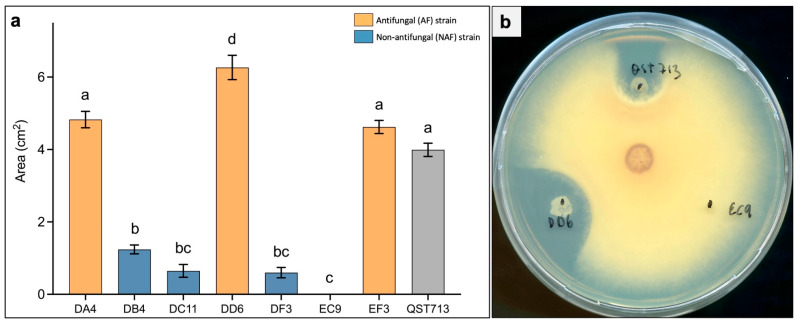
Antifungal activity of bacterial strains against *Fusarium oxysporum in vitro*. (**a**) Antifungal activity measured as the area of fungal growth inhibition between the fungal and bacterial colonies at 7 days after the start of the confrontation test. (**b**) Representative picture of a confrontation plate between *F. oxysporum* and the selected strains DD6 and EC9. QST713 is a *B. velezensis* strain used as benchmark reference. Error bars represent standard error of the mean, *n* = 6. Treatment means marked with different letters are significantly different at *p* ≤ 0.05.

The level of antifungal activity in vitro against *F. oxysporum* was estimated for the seven AF and NAF strains by tracing and quantifying the area of the inhibition zone at 7 days after the start of the confrontation test (Figure 2). A strong inhibitory activity was seen for strains DA4, DD6 and EF3 (termed AF), whereas weak activity was found for DB4, DC11 and DF3 (termed NAF). Strain EC9 did not show any measurable inhibition zone towards *F. oxysporum*, and strong AF activity was also seen for the reference strain QST713 (Figure 2a). From these results, we selected DD6 and EC9 (Figure 2b) as contrasting AF and NAF strains, respectively, for further evaluation.

**Figure 3:**
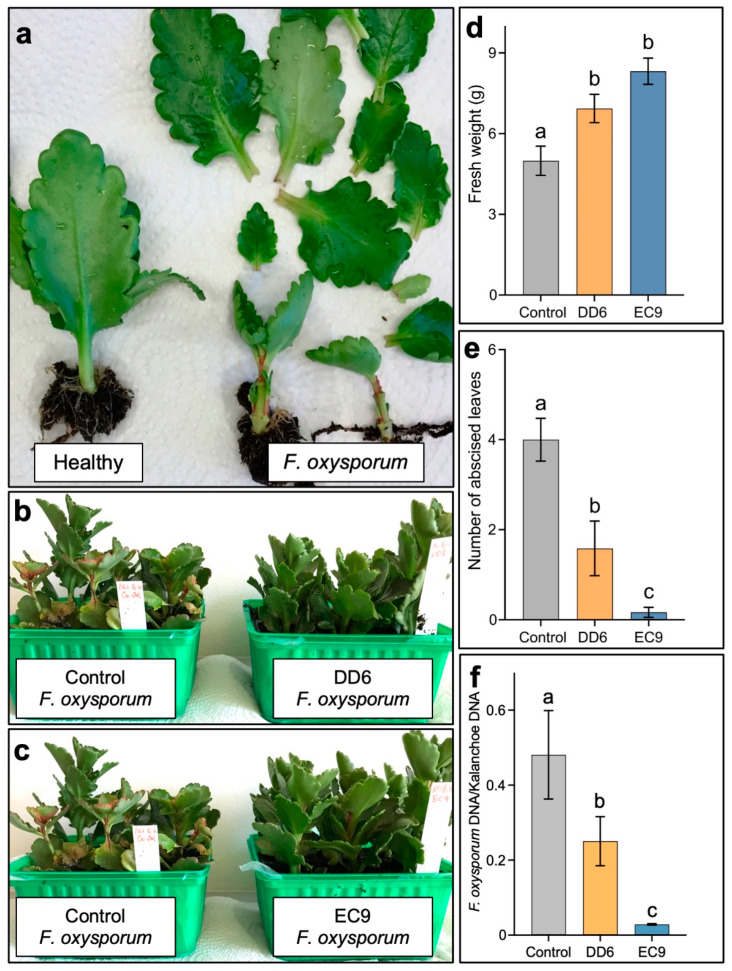
Assessment of the disease protection efficacy of bacterial strains DD6 and EC9 in Kalanchoe plants inoculated with *F. oxysporum*. (**a**) Comparison of a healthy plant (left) with two infected plants (right) 6 weeks after the inoculation showing typical symptoms of wilting, growth retardation and leaf abscission. Disease symptom reduction in plants treated with strain DD6 (**b**) and EC9 (**c**) in comparison to the controls (untreated, inoculated plants). The reduction in disease symptoms was measured indirectly as increase in fresh weight (**d**) and decrease in leaf abscission (**e**) in plants treated with strains DD6 and EC9 (*n* = 12). (**f**) Quantification of *F. oxysporum* DNA in Kalanchoe stems as determined by the ratio of fungal DNA to plant DNA. For each treatment, four groups of three plants pooled together were used for DNA extraction and PCR quantification (*n* = 4). The error bars represent standard error of the mean. Treatment means marked with different letters are significantly different at *p* ≤ 0.05.

DD6 and EC9 were then assessed in greenhouse trials. Here, general disease evaluation was carried out at 6 weeks after inoculation by the visual observation of typical *F. oxysporum* symptoms, including wilting, reduced plant size and leaf abscission (Figure 3a). Treatment with both strains noticeably reduced the symptoms (Figure 3b,c). Disease quantification was subsequently performed indirectly by recording the fresh weight of the above-ground plant parts, by the number of abscised leaves and by the quantification of fungal DNA in the vascular tissue. The results confirmed that both DD6 and EC9 conferred protection against *F. oxysporum*. The fresh weight of plants treated with the two strains was significantly higher than the untreated, inoculated control (Figure 3d). However, interestingly, the NAF strain EC9 protected the plants markedly better than the AF strain DD6. Similarly, even when treatment with the strains resulted in a significant reduction in the number of detached leaves, the reduction in leaf abscission was significantly greater for the NAF strain EC9 (Figure 3e). These results were further supported by the proportion of fungal DNA in the stems of the plants, which showed that only EC9 conferred a significant reduction (Figure 3f).

### 2.4. Whole-Genome Sequencing Analysis Revealed That DD6 and EC9 Belong to Different Species with Distinct Predicted Secondary Metabolite Profiles

**Figure 4:**
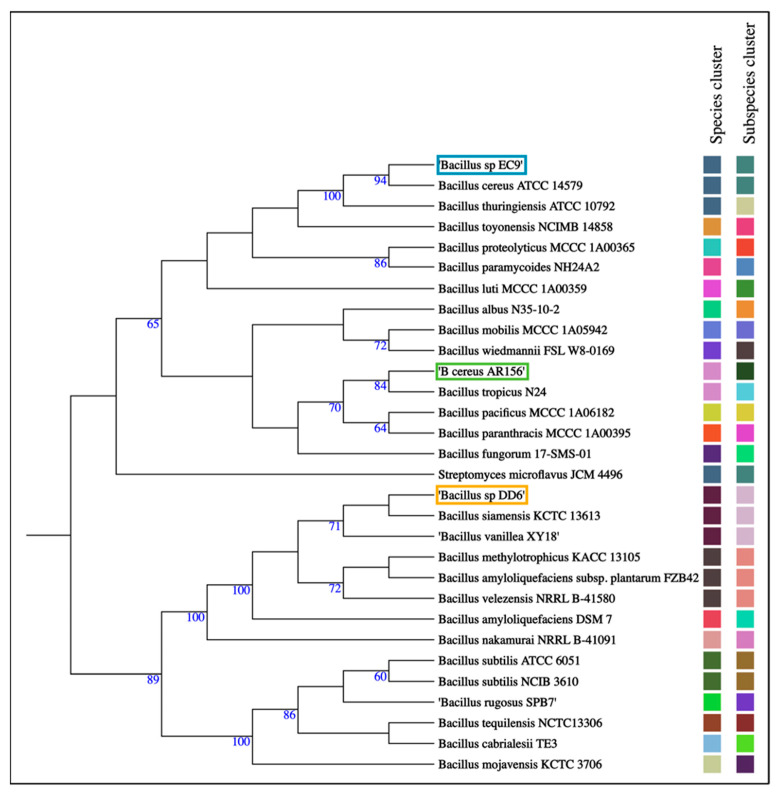
Phylogenetic tree showing grouping of bacterial strains DD6 and EC9, marked in orange and blue boxes, respectively, into species and subspecies clusters. The reference strain *B. cereus* AR156 is marked in a green box. Whole-genome sequences of the strains were analysed by pairwise comparison against a set of best-matching type-strains genome sequences using the Type (Strain) Genome Server (TYGS). Matching square colours indicate same species and subspecies clusters. The tree was inferred according to [35]. The numbers indicate Genome Blast Distance Phylogeny (GBDP) pseudo-bootstrap support values >60% from 100 replications.

Sequencing with Nanopore and Illumina confirmed that DD6 and EC9 are both *Bacillus* strains, but they differ greatly in terms of genome size, C+G content and the presence of two plasmids in EC9 and none in DD6 (Table 1). Genome-based, pairwise comparison indicated that DD6 and EC9 have an average nucleotide identity blast (ANIb) value of approximately 67%, which is well below the cut-off value of 95% used to define species [34] (Table 1). The relationship of the two strains and their respective taxonomic assignment was confirmed by analysis of their complete genomes through the Type (Strain) Genome Server (TYGS) platform [35] (Table 1 and Figure 4). This grouped the strains into separate species clusters, with DD6 clustering at the subspecies level with the reference strains *B. siamensis* KCTC 13613 and *B. vanillea* XY18. Meanwhile, EC9 grouped at the species level with the reference strains *B. thuringiensis* ATCC 10792 and *B. cereus* ATCC 14579, but only with the latter at the subspecies level (Figure 4).

**Figure 5:**
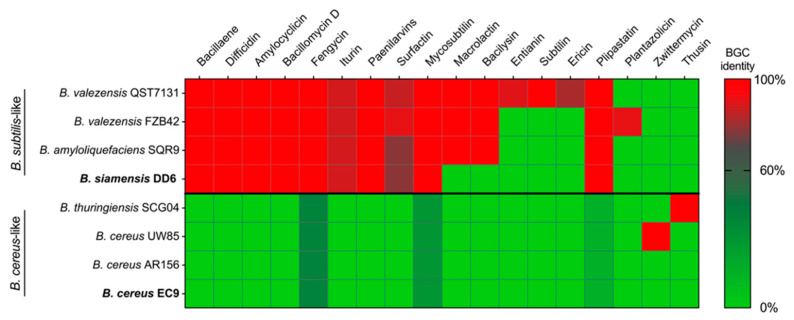
Presence of biosynthetic gene clusters (BGCs) associated with putative, antimicrobial secondary metabolites in bacterial strains DD6 and EC9. Profiling was predicted from the respective whole-genome sequencing using AntiSMASH [37] and compared to a set of genetically related BCA reference strains. The heatmap was constructed using the percentage of sequence identity to BGCs for known antimicrobial metabolites. The cut-off value for presence/absence was set to 60% sequence identity. For details of the reference strains used, see Materials and Methods.

Secondary metabolite profiling predicted from the whole-genome sequencing revealed the presence of several biosynthetic gene clusters (BGCs) potentially involved in antimicrobial activity in DD6, but not in EC9 (Figure 5). Using a cut-off value of 60% BGC sequence identity, ten such BGCs were identified in the AF strain DD6, whereas none were found for the NAF strain EC9. Accordingly, DD6 grouped with the *B. subtilis*-like reference strains *B. amyloliquefaciens* SQR9 and the *B. velezensis* strains, QST713 and FZB42. On the other hand, EC9 lack all the studied antimicrobial BGCs, a feature that it shares with *B. cereus* AR156, while the *B. cereus*-like reference strains *B. thuringiensis* SCG04 and *B. cereus* UW85 each has a single BGC (Figure 5).

### 2.5. DD6 and EC9 Show High Protection Efficacy under Commercial Conditions

**Figure 6:**
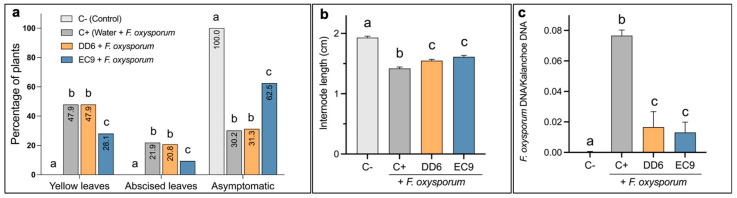
Protection efficacy against *F. oxysporum* infection achieved by treatment with the antifungal DD6 and the non-antifungal EC9 bacterial strains, respectively, in pilot trials under commercial conditions. (**a**) Disease level was assessed at 4 weeks after pathogen inoculation by determining the percentage of asymptomatic plants versus the percentage of plants showing yellow and abscised leaves. The numbers in the bar indicate percentage of plants (*n* = 96). (**b**) Determination of the length of the first two internodes to measure growth retardation (*n* = 96). (**c**) Relative fungal biomass in blocks of 24 plant stems pooled together (*n* = 3). C− denotes untreated, uninoculated control plants, and C+ denotes untreated, inoculated control plants. The error bars represent standard error of the mean. Treatment means marked with different letters are significantly different at *p* ≤ 0.05.

To test their efficacy under commercial conditions, the strains DD6 and EC9 were further evaluated for protection of Kalanchoe against *F. oxysporum* in a pilot trial carried out in the nurseries at Knud Jepsen a/s. In this assessment, the treatment of Kalanchoe cuttings with DD6 and EC9 was performed twice before inoculation, and the symptoms were evaluated at 4 weeks after inoculation by recording the number of plants showing yellowing and abscission of leaves (Figure 6a). In the untreated, inoculated controls (C+), pathogen inoculation resulted in nearly 70% symptomatic plants, where 47.9% showed yellowing of leaves and 21.9% also showed leaf abscission. Treatment with the AF strain DD6 did not result in a significant reduction in symptoms, as opposed to treatment with the NAF strain EC9, which caused a significant reduction in symptoms. Here, 62.5% of the plants remained asymptomatic, and both the number of plants showing yellow and abscised leaves was also significantly reduced (Figure 6a). To evaluate the effect of DD6 and EC9 in preventing plant growth retardation caused by infection, the lengths of the first and second internodes of the plants were measured. Remarkably, this symptom type was reduced by treatment with both strains, i.e., the lengths of the internodes were significantly larger than those of the untreated, inoculated control plants (Figure 6b). Likewise, treatment with both the AF and the NAF strain resulted in strong and significant reductions in the relative amount of fungal DNA in the plant stems (Figure 6c).

### 2.6. Strains DD6 and EC9 Colonise Kalanchoe Roots but Differ in Their Persistence

**Figure 7:**
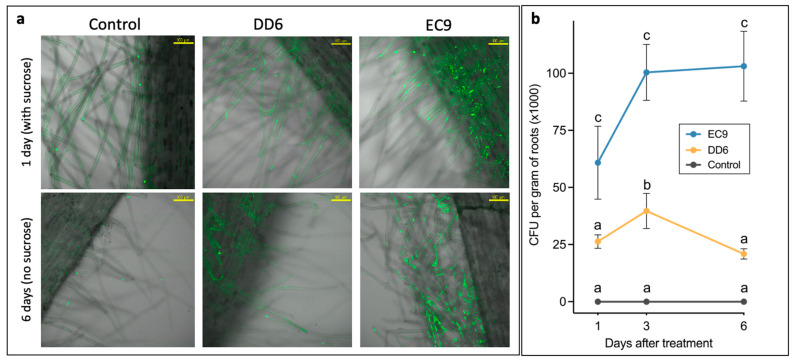
Colonisation of Kalanchoe roots by bacterial strains DD6 and EC9. (**a**) Representative pictures of roots of plants treated under axenic conditions with equal concentrations of the bacterial strains, first in MS medium containing 1% sucrose for 1 day (upper panel) and then for 6 days in MS medium without sucrose (lower panel). The bacterial cells were stained with Syto-13 and visualised by confocal microscopy. The scale bars represent 100 μm. (**b**) Quantification of colonisation persistence by CFU counting at different time points after treatment. All the roots from individual plants were used. Error bars represent standard error of the mean, *n* = 3. Within each time point, different letters indicate significant differences at *p* ≤ 0.05.

We assessed the ability of the two strains to colonise and persist on Kalanchoe roots under axenic conditions (Figure 7). Confocal microscopy indicated that the bacterial cells of both strains attached well to the surface of root hairs at 1 day after treatment. However, observations from distinct sites of root samples from different plants showed that the colonisation of the NAF strain EC9 was more profuse than the AF strain DD6 (Figure 7a). After further incubation without a source of carbon in the medium, a clear reduction in root-colonising bacterial cells was seen for DD6, whereas EC9 showed an extensive colonisation of root hairs after 6 days (Figure 7a). Unspecific staining with no distinct root-colonising bacteria were detected on the controls. To confirm these observations, we quantified the root colonisation ability of the strains by determining the colony-forming unit (CFU) number per gram of root after incubation for 1 day in MS + S medium and 2 and 6 days in medium without sucrose (Figure 7b). Already after 1 day, the number of CFUs for DD6 was significantly lower in comparison to EC9. Although for both strains, the CFU number increased after 3 days, additional incubation resulted in a drop in CFU count for DD6, whereas for EC9, it remained steady after 6 days (Figure 7b). These results confirmed the microscopy observation of EC9 showing a superior ability to colonise and persist on Kalanchoe roots compared to DD6. No CFUs were obtained in the controls.

### 2.7. EC9 Induces Defence Priming against F. oxysporum Infection in Roots

**Figure 8:**
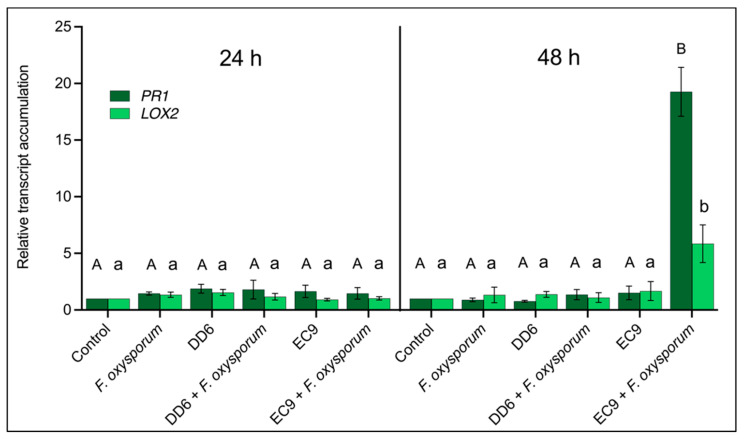
Defence-related gene expression in Kalanchoe roots pre-treated with bacterial strains DD6 or EC9 and subsequently inoculated with *F. oxysporum*, as described in Materials and Methods. Roots were sampled at 24 and 48 h after the inoculation. The expression of *PR1* and *LOX2* transcripts was determined relative to the controls (no bacteria, no *F. oxysporum*). Error bars represent standard error of the mean (*n* = 3). Different letters indicate significant differences at *p* ≤ 0.05. Capitalised and non-capitalised letters refer to statistical differences in the *PR1* and *LOX2* transcript levels, respectively.

Despite EC9 not being antifungal, it conferred better protection against *F. oxysporum* infection than DD6. To determine if the protection could be due to augmented immunity against fungal attack in bacteria-treated Kalanchoe roots, we tested whether the salicylic acid (SA) and jasmonic acid (JA) signalling pathways are activated. *PR1* and *LOX2* encoding pathogenesis-related protein 1 and lipoxygenase 2, respectively, are commonly used marker genes for these respective pathways [38], and we retrieved the *Kalanchoe fedtschenkoi* sequences of these genes (accession numbers Kaladp0099s0143.1 and Kaladp0747s0006.1) by BLAST using *Arabidopsis thaliana PR1* (AT2G14610) and *LOX2* (AT3G45140) in Phytozome V13 [39]. This allowed us to perform *PR1* and *LOX2* quantitative RT-PCR in plants pre-treated with DD6 and EC9 followed by subsequent inoculation with *F. oxysporum*. At 24 h after fungal inoculation, neither treatments with each of the two bacterial strains, inoculation with pathogen nor the combinations of these changed the transcript levels of *PR1* and *LOX2* in roots (Figure 8). However, at 48 h after pathogen inoculation, there was a significant increase in the levels of both transcripts, but only in the EC9-treated, *F. oxysporum*-inoculated roots (Figure 8). Here, there was a 20-fold induction for *PR1* and a 5-fold induction for *LOX2*. No significant difference in gene expression was observed in the roots after the remaining treatments. The upregulation of these defence-related transcripts only occurred in EC9-treated roots that were later inoculated with *F. oxysporum*, suggesting that EC9 primes the roots for enhanced immunity following a subsequent pathogen attack.

## 3. Discussion

Biofungicides are a relevant alternative to reduce the dependence of agriculture on synthetic fungicides [11]. In this approach, antibiosis has been considered as a recognisable mode of action of BCAs exploited to directly target fungal pathogens [11,13]. Rhizobacteria, particularly *Bacillus* sp., are an important source of BCAs producing fungitoxic compounds [12,40,41]. Yet, the utilisation of beneficial microorganisms to target the plant immune system has not received the same attention [42]. In this study, we tested endospore-forming bacteria sampled from waste material from Kalanchoe cultivation and, by combining conventional in vitro antifungal screening with in planta evaluation, we identified a number of *Bacillus* strains that provided protection to Kalanchoe against *F. oxysporum*, a recognised soil-borne pathogen, penetrating through the roots and invading the vascular tissue [43]. Notably, several of these protecting strains did not show antifungal activity in vitro. The selection and evaluation of two strains, DD6 and EC9, which are contrasting in terms of in vitro antifungal activity (Figure 2), provided insights into their efficacy *in planta* and the possible mechanisms responsible for protection. Although both strains showed high protection efficacy under experimental setups, the NAF strain EC9 showed superior protection (Figure 3). The same pattern of protection was observed in pilot trials under commercial conditions (Figure 6). In the latter case, the trial was set up to mimic a commercial plant production process, where the bacteria were applied through sub-irrigation, requiring that the bacteria suspended in water reached the roots from below. Like in the experimental setup, the commercial setups also showed that both DD6 and EC9 resulted in significant increases in plant size (Figure 6b) and a decrease in fungal DNA in the stems (Figure 6c). However, only treatment with the NAF strain EC9 caused a sufficient decrease in symptomatology, measured as leaf yellowing and abscission (Figure 6a), to increase the commercial value of Kalanchoe plants. We further confirmed the non-antifungal activity of EC9 in vitro as well as its ability to provide protection to tomato against the wilt pathogen, *F. oxysporum* f.sp *lycopersici* (unpublished results). Interestingly, these results suggest that EC9 protects by a mechanism distinct from antibiosis, possibly through modulation of the plant immune system (see below).

Whole-genome sequencing revealed that DD6 and EC9 belong to distinct phylogenetic groups within the Bacilli (Figure 4, Table 1). Here, the AF strain DD6 clustered within the *B. subtilis*-like group, whereas the NAF strain EC9 grouped with the *B. cereus sensu lato* group [44]. The *B. subtilis*-like group contains many of the well-known species with antimicrobial properties [40], and DD6 shares several BGCs that potentially mediate such properties with the reference species *B. amyloliquefaciens* and *B. velezensis* (see below). On the other hand, EC9 is related to species such as *B. cereus* and *B. thuringiensis*. Additionally, the genome size, G + C content and plasmid content of DD6 and EC9 differ significantly (Table 1). Further analysis using antiSMASH prediction suggests that DD6 and EC9 have very different secondary metabolite profiles, as inferred from the presence of BGCs potentially involved in antimicrobial activity (Figure 5). The strong antifungal activity in vitro of strain DD6 towards *F. oxysporum* (Figure 2) can be linked to the presence of several BGCs in the bacterial genome that are associated with recognised antimicrobial metabolites. For example, DD6 contains many of the BGCs present in the BCA strains *B. velezensis* QST713 and FZB42 and *B. amyloliquefaciens* SQR9, which utilise an array of antimicrobial metabolites, including fengycins, bacillomycins, surfactins and iturins, to antagonise fungi [17,41,45,46]. We further established the contrasting antifungal activity in vitro of strains DD6 and EC9 for the tomato wilt pathogen *F. oxysporum* f.sp *lycopersici* and the tomato crown and root rot pathogen *F. oxysporum* f.sp. *radicis-lycopersici* (unpublished results).

EC9 diverges significantly from the reference strains of the *Bacillus subtilis*-like group shown in Figure 5 by the absence of BGCs related to typical antifungal compounds. This lack of BGCs is shared with other members of the *Bacillus cereus sensu lato* group such as *B. thuringiensis*, which is one of the species most widely used as a BCA, particularly for its insecticidal activity [47,48]. It should be noted that antifungal activity has also been reported for some strains of *B. thuringiensis* [49,50]. The strain EC9 is related to *B. cereus* at the phylogenetic and secondary metabolite profile levels (Figure 4 and Figure 5). Although *B. cereus* is commonly referred to as a bacterium causing food poisoning [51], several strains of this species have BCA properties [52]. For example, *B. cereus* UW85 suppresses damping-off caused by the oomycete *Phytophthora megasperma* in alfalfa through the antimicrobial compound zwittermicin A [53] (Figure 5). In terms of the absence of antifungal-compound-related BGCs, the BCA strain in the *B. cereus sensu lato* group most similar to EC9 is AR156 (Figure 5). However, the ANIb and phylogenetic analyses showed that AR156 differs from EC9 by genome size and number of plasmids (Table 1), grouping them in separate species and subspecies clusters (Figure 4). Notably, AR156 also induces defence pathways in *Arabidopsis thaliana* [54,55]. In tomato, the presence of root exudates positively influences root colonisation and biocontrol activity of AR156 against *Ralstonia solanacearum* [56].

To investigate whether the strains DD6 and EC9 can influence plant defence responses, we studied their ability to colonise and persist on Kalanchoe roots under axenic conditions. Since root exudates are crucial for successful stable colonisation [56,57,58], we tested the ability of DD6 and EC9 to colonise and survive solely from root exudates (Figure 7a). Following microscopy, Syto-13-stained DD6 and EC9 bacterial cells were observed attached to roots and root hairs of Kalanchoe at 1 day after treatment, indicating positive initial colonisation. However, incubation for 6 days in the absence of sucrose showed a drastic reduction in root-attached cells of DD6, whereas cells of EC9 exhibited extensive and overlapping colonies on root hairs (Figure 7a). This observation was supported by CFU quantification of living bacteria on the roots, where EC9 showed better root colonisation performance (Figure 7b). Thus, the superior ability of the NAF strain EC9 to colonise and persist on Kalanchoe roots can contribute to its ability to stimulate the plant immune system. To investigate this, we studied the expression of the marker genes, *PR1* and *LOX2*, that in several species are associated with the salicylic acid and jasmonic acid defence pathways, respectively [38]. We saw significantly increased transcript levels of *PR1* and *LOX2* only when roots were treated with EC9 and 48 h after inoculation with *F. oxysporum* (Figure 8). Notably, treatment with EC9 alone did not result in changes in the transcript levels. On the other hand, the AF strain DD6, neither alone nor in combination with the pathogen, triggered defence marker transcript accumulation (Figure 8), despite the fact that antimicrobial compounds, such as surfactin and fengycin potentially produced by DD6 (Figure 5), have been reported to activate plant immunity [59]. The SA and JA defence pathways are frequently regarded as mutually antagonistic and to be specifically activated by biotrophic and necrotrophic pathogens, respectively [21,22,60,61]. Additionally, triggering of ISR by plant-growth-promoting rhizobacteria (PGPR) has been considered to be mediated primarily by JA-related defence pathways [62,63]. However, the simultaneous activation of SA and JA responses has been previously reported [64,65]. The concomitant activation of SA- and JA-associated defence responses has, for instance, been found in the rhizobacterial-mediated activation of defence priming [27,28]. Similarly, our transcript expression data suggest that the NAF strain EC9 triggers the simultaneous activation of SA and JA defences, but only after challenge with *F. oxysporum* (Figure 8). The fact that only the combination of EC9 and *F. oxysporum* activates defence response transcript accumulation suggests that the protection involves defence priming. This is similar to *B. cereus* strain AR156 in *A. thaliana*, where the activation of SA and JA defences was shown to be faster and stronger following challenge with *Pseudomonas syringae* pv. *tomato* and was suggested to indicate a defence-priming effect [54].

Despite the fact that the genomic data indicate that EC9 and AR156 are distinct species within the *B. cereus sensu lato* group (Figure 4), they both lack BGCs associated with antifungal activity in their genome (Figure 5). Furthermore, they share the ability to confer protection against pathogens through the activation of defence priming. In line with our Kalanchoe data for EC9, we also found an upregulation of the SA-marker transcript for isochorismate synthase and a JA-marker transcript for a proteinase inhibitor in tomato plants protected from *F. oxysporum* f.sp *lycopersici* infection by treatment with EC9 (unpublished results).

The application of induced resistance for crop disease protection has been considered for a long time [42]. For instance, the synthetic resistance inducer benzothiadiazole represented a breakthrough in this field [66]. However, a major drawback of many biotic and abiotic elicitors of induced resistance has been the supposed energy costs for the plant associated with the long-term activation of resistance in the absence of infection [67]. Contrastingly, priming of defence, which can be activated biotically and abiotically without energy costs for the plant, emerges as a promising approach for crop protection [32]. Thereby, more systemic and longer-lasting protection potentially mediated by non-antifungal BCAs, such as EC9 and AR156, becomes more attractive. Future studies should compare the protection efficacies of EC9 and AR156, including how and to what extent these *B. cereus* strains prime defence on different plant species, since they were identified from different sources.

The screening and identification of BCAs with alternative modes of action require detailed studies on the interplay between the BCA, the pathogen and the host plant. Deeper studies of mechanistic aspects responsible for the protective effect provided by EC9 are currently ongoing in tomato and Arabidopsis. Furthermore, studies of how the environment impacts this tripartite interaction under field conditions are required. The suitable development of crop protection products based on plant-defence-inducing BCAs, including large-scale production and formulation, is also needed.

In this study, we found that stimulation of the plant immune system mediated by BCAs can make a contribution to crop protection that is comparable to the antifungal approach. Appealingly, the combination of BCAs with complementary modes of action, such as DD6 and EC9, may result in synergistic protection efficacy, which opens new possibilities for developing biological crop protection products with superior efficacy.

## 4. Materials and Methods

### 4.1. Sampling and Isolation of Endospore-Forming Bacteria from Kalanchoe-Associated Material

Samples of discarded plant and coconut peat substrate waste, previously used for cultivating Kalanchoe, were obtained from Knud Jepsen a/s’ commercial nurseries. One gram of air-dried sample material was ground using a mortar and pestle and transferred into a sterile 15 mL centrifuge tube. Ten millilitres of sterile saline water (0.9% NaCl, 0.01% Tween 20 (Merck, Darmstadt, Germany)) was added, and the tube was vortexed for 10 min. One millilitre of sample suspension was transferred to a sterile 1.5 mL centrifuge tube and incubated for 20 min at 80 °C with continuous agitation. A series of 1:10 dilutions were prepared in 0.9% NaCl, and 100 µL of each dilution was distributed onto Luria–Bertani (LB) agar (LBA) plates using a T-shaped spreader and incubated at 28 °C for 48 h. Distinct, non-overlapping bacterial colonies were picked and subsequently streaked separately on new LBA plates. After incubation at 28 °C for 48 h, a single colony from each streaked LBA plate was picked and transferred into individual wells of a 2 mL 96-DeepWell Plate (Nunc, Thermo Fisher Scientific, Waltham, MA, USA) containing 500 µL of LB, covered with breathable sealing tape (Nunc, Thermo Fisher Scientific, Waltham, MA, USA) and cultured at 28 °C at 160 rpm for 48 h. Then, 500 µL of 50% sterile glycerol was added to each well and thoroughly mixed, and 300 µL from each well was transferred into individual wells of a 0.5 mL 96-well MicroWell plate (Nunc, Thermo Fisher Scientific, Waltham, MA, USA), covered with a sterile silicone cap mat and stored at −80 °C. Before use, the bacterial strains were recovered from the glycerol stocks and grown in 5 mL culture tubes containing 2 mL of LB. Following overnight incubation at 28 °C with shaking at 160 rpm, 100 µL of the bacterial cultures was distributed on LBA plates. The plates were incubated for 48 h at 28 °C and the bacteria were used for in vitro screening and plant treatments.

### 4.2. In Vitro Screening for Antifungal Activity

The antifungal activity of the bacterial strains was tested on confrontation plates against the *F. oxysporum* isolate CP2321 previously isolated from infected Kalanchoe plants [33]. The fungus was grown on potato dextrose agar (PDA) plates at 28 °C for 7 to 10 days, and agar plugs were taken from the growing edge of the colonies using a 0.5 cm cork borer. A plug of the fungus was placed on the centre of a new PDA plate with the mycelium growth facing down and incubated at 28 °C for 24 h. Subsequently, the fungus was confronted with four different bacterial strains per plate by placing 3 µL of bacterial LB culture, adjusted to OD_600_ = 0.3, at 2 cm from the agar plug with the fungus. The plates were incubated at 28 °C for 5 to 10 days depending on the growth rate of the bacteria. AF and NAF strains were preliminarily recognised by the presence or absence of clear growth-inhibition zones, respectively. Randomly selected NAF strains were also preliminarily tested *in planta* using the procedure described below. Subsequently, in vitro antifungal activity of selected strains was quantified at seven days after the start of confrontation by tracing and measuring the area of the inhibition zone around the bacterial colonies of six independent plates, using the ImageJ software [68]. *Bacillus velezensis* strain QST713, which is the active ingredient of the biological control product Serenade ASO (Bayer Crop Science, Leverkusen, Germany), was used as a reference strain.

### 4.3. Preparation of Bacterial Suspensions and Treatment of Kalanchoe

Single bacterial colonies were taken from overnight LBA plates, cultured for 48 h in 50 mL of LB medium at 28 °C with shaking at 160 rpm. Subsequently, the cultures were centrifuged at 4000× *g* for 10 min, the cells were washed once with 10 mM MgCl_2_, resuspended in the same buffer, adjusting the concentration to OD_600_ = 0.3.

The experiments were carried out in the greenhouse at 22/18 °C, 16/8 h day/night with supplementary daylight of 160 μmol m^−2^ s^−1^ using the *F. oxysporum*-susceptible Kalanchoe cultivar Margrethe [33]. Rootless cuttings were planted in vermiculite and grown for 10–12 days until newly developed roots were 0.5–1 cm. The rooted cuttings were then pulled out, and the remaining vermiculite was rinsed off the roots under deionised water. Subsequently, the cuttings were treated with each bacterial strain by immersing the roots into a sterile plastic box containing 100 mL of bacterial suspension for 60 min at room temperature with constant shaking at 90 rpm. Control cuttings were treated with 10 mM MgCl_2_. After treatment, the cuttings were directly planted in substrate (commercial peat moss, Pindstrup Substrate no. 2, Pindstrup Mosebrug, Ryomgård, Denmark) and grown for three days in the greenhouse before pathogen inoculation.

### 4.4. Pathogen Inoculation

Fungal spore suspensions of *F. oxysporum* were prepared by adding 10 mL of distilled sterile water to 3-week-old PDA cultures and thoroughly scraping the surface with a sterile plastic spatula. The spore suspension was filtered through three layers of gauze, and the concentration was adjusted to 5 × 10^5^ spores/mL. The bacteria-treated cuttings were carefully pulled out from the substrate and dip-inoculated by immersing the roots, including adhered substrate, into a sterile plastic box containing 100 mL of *F. oxysporum* spore suspension for 60 min with constant shaking at 90 rpm. Controls were treated in the same way in sterile water. After inoculation, the cuttings were re-planted in the same pots and cultivated in the greenhouse for 6 weeks. The level of disease was evaluated by at least two of the following criteria: (1) fresh weight of the above-ground part of the plant; (2) leaf abscission measured as the number of leaves that detached from the plant following strong manual shaking; (3) estimation of the relative amount of fungal DNA (see below); and (4) the extent of the area showing vascular necrosis (VN) in cross-sections of stems at the crown level using the following scores: 0 = no VN, 1 = 1–25% VN, 2 = 26–50% VN, 3 = 51–75% VN and 4 = 76–100% VN.

### 4.5. Quantification of the Relative Amount of F. oxysporum in Kalanchoe Stems

The extraction of plant and fungal DNA, primers and quantitative PCR conditions was performed as previously described [33]. Briefly, the amount of fungal DNA was estimated by calculating the ratio of fungal DNA to Kalanchoe DNA using serial dilutions from 0.001 to 10 ng of pure genomic DNA from each organism. Standard curves were fit by linear regression, and the amount of DNA was estimated by tracing the Ct-values against the known amount of DNA [69].

### 4.6. Whole-Genome Sequencing and Prediction of Secondary Metabolite Biosynthesis Gene Clusters

Selected bacterial strains were cultured for 72 h in LB as described above, and the genomic DNA was extracted using the Genomic Mini Bacteria kit from A&A Biotechnology (Gdansk, Poland). DNA quality and quantity were checked on a Nanodrop ND-1000 spectrophotometer and Qubit 2.0 fluorometer (Thermo Fisher Scientific, Waltham, MA, USA). Libraries for Nanopore sequencing were prepared using the Rapid Barcoding Sequencing kit (SQK-RBK004) and sequenced on the MinION platform using MinKNOW (v4.1.22) (all from Oxford Nanopore Technologies, Oxford, UK). Base calling of raw reads was performed with the Guppy base calling software (v4.2.2) (Oxford Nanopore Technologies, Oxford, UK), using the high-accuracy model. Adapter sequences were trimmed from Nanopore reads using Porechop (v0.2.4) [70]. For Illumina sequencing, libraries were prepared using the Nextera XT library kit and sequenced on the NextSeq 550 platform with a Mid Output Kit v2.5 (300 cycles) (Illumina Inc. San Diego, CA, USA). Adapter sequences and barcodes were trimmed from Illumina reads using Trim Galore [71] (v0.6.4). Hybrid assemblies were performed with Unicycler [72] (v0.4.8) and genomes were annotated with Prokka [73] (v1.14.6). Plasmids were characterised according to type of mobilisation and relaxed-typing, using MOB-suite [74] (v3.0.0). The assembled genome sequences were analysed for their mutual relatedness using the average nucleotide identity blast (ANIb) tool of the JSpeciesWS platform [34]. The sequences were uploaded to the Type (Strain) Genome Server (TYGS) for whole-genome-based taxonomic analysis [35] through pairwise comparisons against a set of best-matching type-strain genome sequences to infer a phylogenetic tree with species and subspecies clustering. Secondary metabolite profiling and the prediction of biosynthetic gene clusters (BGCs) were performed with the Antibiotic and Secondary Metabolite Analysis Shell (AntiSMASH 6.0) [37]. The presence of specific BGCs possibly involved in the production of several known antimicrobial metabolites [13,16,17] was compared to the following *Bacillus* reference strains with recognised or potential use as BCAs: *B. velezensis* QST713 [75], *B. velezensis* FZB42 [76], *B. amyloliquefaciens* SQR9 [77], *B. thuringiensis* SCG04 [48], *B. cereus* UW85 [53] and *B. cereus* AR156 [36].

### 4.7. Pilot Study under Commercial Conditions

The selected antifungal (AF) and non-antifungal (NAF) strains DD6 and EC9, respectively, were evaluated in a pilot study carried out at the Knud Jepsen a/s nurseries, Hinnerup, Denmark. The Kalanchoe cultivar “Margrethe”, susceptible to *F. oxysporum*, was used. Cuttings were planted individually in 6 cm pots containing commercial peat moss (Pindstrup Special Mix, Pindstrup Mosebrug, Pindstrup, Denmark) and grown at 19/21 °C, 10/14 h day/night with supplementary daylight of 80 μmol m^−2^ s^−1^. During the experiment, sub-irrigation was applied two to three times per week and supplemented with micro and macronutrients according to a standard irrigation and fertilisation programme of the company. Combinations of the following were performed: treatment with the AF strain DD6, the NAF strain EC9 or mock (water); inoculation with *F. oxysporum* or uninoculated control. For each treatment, 96 plants were used. One week after planting of cuttings, the treatment with the bacterial strains was performed at 7 and 4 days before pathogen inoculation. The bacterial strains were previously cultured in LB as described above and mixed with the irrigation water to a final OD_600_ of 0.2. Treatment was performed by sub-irrigation of the plants with a flooding period of 10–15 min. Inoculum of *F. oxysporum* was prepared from PDA cultures as described above. Immediately before inoculation, the roots were manually wounded by briefly uprooting and re-planting. Ten millilitres of inoculum was applied around the base of the plants. Disease evaluation was carried out 4 weeks after inoculation using the following three parameters: (1) percentage of plants showing leaf yellowing and abscission; (2) plant growth retardation assessed by measuring the length of the first two internodes from the base of the plant; and (3) relative fungal DNA in the stems using three blocks of 24 plants that were sampled and pooled together. For each treatment, three blocks were analysed independently using the qPCR procedure described above.

### 4.8. Root Colonisation Studies

Rootless Kalanchoe cuttings were surface sterilised for 4 min in 2% sodium hypochlorite, 5 min in 70% EtOH and rinsed five times for 1 min in sterile deionised water. The cuttings were placed individually into 50 mL sterile plastic vials containing 10 mL of sterile ½ x Murashige and Skoog medium (Duchefa Biochemie BV, Haarlem, The Netherlands) supplemented with 1% (*w*/*v*) sucrose (MS + S), allowing the base of the cutting to be submerged into the medium. The vials were placed in the growth chamber for 7–10 days with 14 h of light at 200 μmol m^−2^ s^−1^ with continuous shaking at 140 rpm, until the roots were 1–2 cm long. Three vials were used for each treatment. The AF and NAF strains DD6 and EC9, respectively, were grown as described above and resuspended in MS + S to OD_600_ = 0.1. For treatment of the cuttings, the MS + S medium was discarded and replaced with fresh MS + S medium containing the bacteria. MS + S without bacteria was used as control. After 1 day, three to five single roots were sampled from each treatment and placed in a sterile 1.5 mL tube, rinsed twice with phosphate-buffered saline (PBS, pH 7.4) and subsequently fixed with 4% formaldehyde in PBS. The cuttings were then returned to the vials, and the MS + S medium containing bacteria was replaced with fresh sterile 0.5 X MS without sucrose (MS-S) and further incubated for 6 days. Then, additional roots were sampled and fixed as before. Following fixation, the roots were rinsed twice in PBS and stained with 5 μM Syto-13 green-fluorescent nucleic acid stain (Molecular Probes, Life Technologies, Carlsbad, CA, USA) in PBS for 10 min in the dark. The staining solution was replaced with sterile water, and the samples were observed in a Leica SP5X confocal microscope (Leica Microsystems, Wetzlar, Germany) using 488 nm excitation Argon laser and 506 nm emission. To quantify the colonisation of the bacteria on the roots, a similar experimental setup was carried out. In this case, all the roots from each plant were harvested at 1, 3 and 6 days, rinsed once in MS-S, weighed, and thoroughly ground in the same medium before determination of the number of colony-forming units (CFUs) on LBA plates.

### 4.9. Defence-Related Gene Expression in Kalanchoe Roots

The activation of plant defence responses was determined in Kalanchoe roots following treatment with the strains DD6 and EC9 alone or after inoculation with *F. oxysporum*. The preparation of bacterial and fungal spore suspensions and concentrations was carried out as described above. Rootless cuttings were grown in vermiculite for 10 days in the growth chamber at 25/20 °C, 12/12 h day/night with daylight of 200 μmol m^−2^ s^−1^. The cuttings were then treated at 7 and 2 days before the inoculation by drenching 10 mL of the bacterial suspension around each plant. Pathogen inoculation was performed by drenching 10 mL of spore suspension. Combinations of the following were performed: treatment with the AF strain DD6, the NAF strain EC9 or mock (water); inoculation with *F. oxysporum* or uninoculated control. A total of 18 plants, divided in three blocks of six plants, were used for each treatment. For each block, half of the plants were harvested at 24 h and the other half at 48 h after the inoculation. For each time-point, the roots cut from each plant were pooled and immediately frozen in liquid nitrogen. Total RNA was extracted using the Monarch Total RNA Miniprep Kit (New England Biolabs, Ipswich, MA, USA). The RNA was used as a template for first-strand cDNA synthesis using the ProtoScript II kit (New England Biolabs, Ipswich, MA, USA). Real-time RT-PCR was performed according to the 2^−ΔΔC^_T_ method [78] using the Hot FirePol EvaGreen kit (Solis Biodyne, Tartu, Estonia) and the LightCycler96 System (Roche Diagnostics GmbH, Rotkreuz, Switzerland) according to the PCR profile recommended by the manufacturers. The gene-specific primer pairs KalPR1-3 (forward: 5′-AACATCGCTATGTCCACGGG-3′ and reverse: 5′-CCCCAAGCGAGTCGAGTTAG-3′ for Kaladp0099s0143.1) and KalLOX2-1 (forward: 5′-TCGCAAAAACATTCCCAGCG-3′ and reverse: 5′-AGCGCTATCTCTGGCTTGTC-3′ for Kaladp0747s0006.1) [39] were designed using NCBI Primer Designing Tool [79]. The primer pair KdActin used for PCR normalisation was obtained for the *Actin* gene of *Kalanchoe daigremontiana* [80]. PCR optimisation and efficiency calculation was carried out using 1:10 standard curves of known DNA concentrations. The upregulation of *PR1* and *LOX2* was confirmed by treatment of Kalanchoe roots with salicylic acid and methyl jasmonate, respectively (Figure S1). All PCR reactions were performed using three technical replications of each biological replication, and a high-resolution melting analysis was performed to confirm single-product amplification.

### 4.10. Statistical Analyses

All analyses were performed in PC-SAS (release 9.4, SAS Institute, Cary, NC, USA). Hypotheses were rejected at *p* < 0.05. Data disease scoring of vascular necrosis, the quantification of fungal DNA, in vitro antifungal effects, plant fresh weight, internode length and CFU counts were analysed by a mixed-effect model analysis of variance, with treatment (bacterial strain) as the fixed effect and plant (or Petri dish for the in vitro experiment) as the random effect, using the procedure Proc Mixed. Data for the number of abscised leaves under controlled conditions represent a discrete variable and were therefore analysed by logistic regression, assuming a binomial distribution and analysed by a mixed-effect model analysis of variance, with treatment (bacterial strain) as the fixed effect and plant as the random effect using the procedure Proc Glimmix. Data from the pilot study under commercial conditions also represent discrete variables, but the experimental design made it necessary to analyse data by Fisher’s exact test (Proc Freq).

## Figures and Tables

**Table 1:** Genome characteristics and taxonomic species assignment of selected strains.

Strain	Genome Size (bp)	G + C (%)	Mutual ANIb (%) ^1^		Plasmids (bp)	Taxonomic Assignment
			DD6	EC9		
DD6	3,930,487	46.1	-	66.73	None	*B. siamensis*
EC9	5,363,515	35.0	66.73	-	513,295–54,053	*B. cereus*
AR156 ^2^	5,671,798	35.5	66.75	91.42	459,971–40,712–10,789	*B. cereus*

^1^ Average nucleotide identity blast. ^2^ Reference strain *Bacillus cereus* AR156 [36].

## Data Availability

The data that support the findings of this study are available from the corresponding author upon request.

## Supplementary Materials

The following supporting information can be downloaded at: https://www.mdpi.com/article/10.3390/plants11050687/s1, Figure S1: Upregulation of the defence-related genes *PR1* and *LOX2* in Kalanchoe roots by treatment with SA and MeJA.
