# Supplementary material for: The Bacillus cereus Strain EC9 Primes the Plant Immune System for Superior Biocontrol of Fusarium oxysporum
Source: Plants (Basel). 2022 Mar 2;11(5):687. doi: 10.3390/plants11050687 (PMC8912794; doi:10.3390/plants11050687)
Supplement: Supplementary file 1 [file plants-11-00687-s001.zip › plants-1620755-supplementary.pdf]

plants

Article

The *Bacillus cereus* Strain EC9 Primes the Plant Immune System for Superior Biocontrol of *Fusarium oxysporum*

Madriz-Ordeñana et al.

Supplementary Figure S1

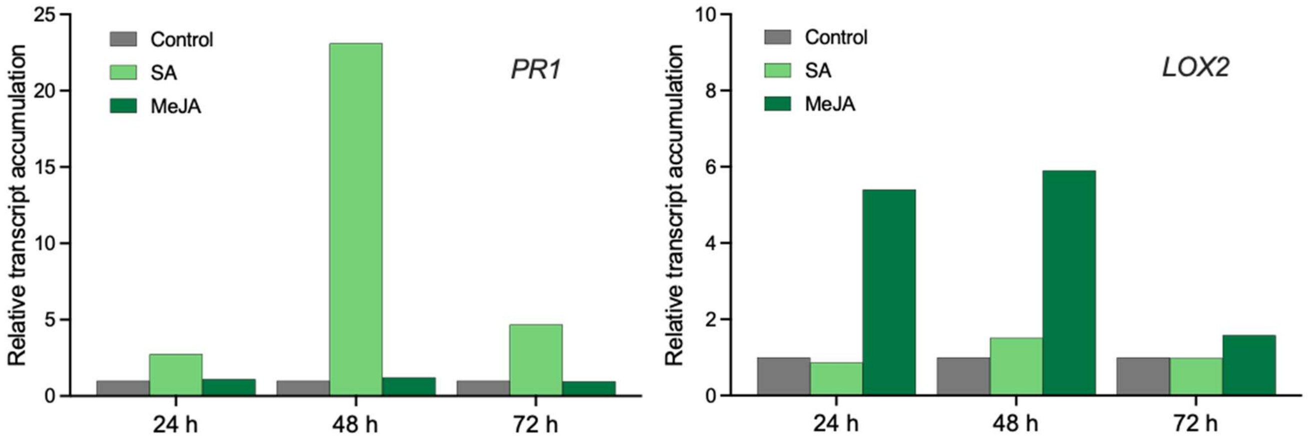

Supplementary Figure S1. Upregulation of the defence-related genes *PR1* and *LOX2* in Kalanchoe roots by treatment with SA and MeJA. Rootless cuttings were grown in vermiculite for 21 days in the growth chamber at 25/20 °C, 12/12 h day/night with daylight of 150 µmol m^{-2} s^{-1}. The roots were treated by drenching fifteen millilitres of 100 µM salycilic acid (SA) or 100 µM methyl jasmonate (MeJA). Roots were harvested at 24, 48 and 72 h after treatment and immediately frozen in liquid nitrogen. RNA extraction, cDNA synthesis and real time RT-PCR was done as described in materials and methods using the *PR1* and *LOX2* specific primers KalPR1-3 and KalLOX2-1, respectively, and the KdActin primer pair for PCR normalization. Transcript accumulation was determined relative to the controls (treatment with water).
